# Apical amplification—a cellular mechanism of conscious perception?

**DOI:** 10.1093/nc/niab036

**Published:** 2021-10-13

**Authors:** Tomáš Marvan, Michal Polák, Talis Bachmann, William A Phillips

**Affiliations:** Department of Analytic Philosophy, Institute of Philosophy, Czech Academy of Sciences, Jilská 1, Prague 110 00, Czech Republic; Department of Philosophy, University of West Bohemia, Sedláčkova 19, Pilsen 306 14, Czech Republic; School of Law and Cognitive Neuroscience Laboratory, University of Tartu (Tallinn branch), Kaarli pst 3, Tallinn 10119, Estonia; Faculty of Natural Sciences, University of Stirling, Stirling FK9 4LA, UK

**Keywords:** apical amplification, perceptual consciousness, perceptual content-specific activity, unconscious content-specific processing

## Abstract

We present a theoretical view of the cellular foundations for network-level processes involved in producing our conscious experience. Inputs to apical synapses in layer 1 of a large subset of neocortical cells are summed at an integration zone near the top of their apical trunk. These inputs come from diverse sources and provide a context within which the transmission of information abstracted from sensory input to their basal and perisomatic synapses can be amplified when relevant. We argue that apical amplification enables conscious perceptual experience and makes it more flexible, and thus more adaptive, by being sensitive to context. Apical amplification provides a possible mechanism for recurrent processing theory that avoids strong loops. It makes the broadcasting hypothesized by global neuronal workspace theories feasible while preserving the distinct contributions of the individual cells receiving the broadcast. It also provides mechanisms that contribute to the holistic aspects of integrated information theory. As apical amplification is highly dependent on cholinergic, aminergic, and other neuromodulators, it relates the specific contents of conscious experience to global mental states and to fluctuations in arousal when awake. We conclude that apical dendrites provide a cellular mechanism for the context-sensitive selective amplification that is a cardinal prerequisite of conscious perception.

## Introduction

More than a decade ago, John Bickle announced the coming era of “molecular and cellular consciousness studies” (Bickle 2007, 291). Whereas mainstream cognitive neuroscience usually investigates consciousness at network and higher levels, cellular and molecular approaches to consciousness are concerned with processes within individual neurons. Although this low-level approach is not yet common in consciousness science (for notable recent exceptions, Flohr 2000; see Sevush 2016; Laberge and Kasevich 2007; Aru *et al.* 2020b), it has several strengths to recommend it. Cellular approaches to consciousness, and to cognition in general, build upon decades of successful neurobiological work and proceed by the highly effective methods of precise measurements and targeted interventions into the cellular processes.^1^

In this article, we aim to contribute to this nascent and exciting field of study. Anesthesia (Suzuki and Larkum 2020), absence epilepsy (Cossart *et al.* 2001; Kole *et al.* 2007), and slow-wave sleep (Aru *et al.* 2020a) all prevent apical input to neocortical cells amplifying their output, so we consider the possibility that conscious experience when awake depends on apical amplification (AA). The theory of conscious perception presented here aims to explain how mechanisms within a particular class of pyramidal neurons, i.e. context-sensitive cells in layer 5B of the neocortex, provide cellular foundations for network-level processes that have long been thought to be neuronal correlates of perceptual experience. We start with a short overview of the mechanisms and then guide the reader through four distinctive features of the theory. The ways in which this article complements and advances beyond previous articles on the perspective that we advocate are listed in the “Concluding remarks” section.

## The mechanism of AA

**Figure 1.**
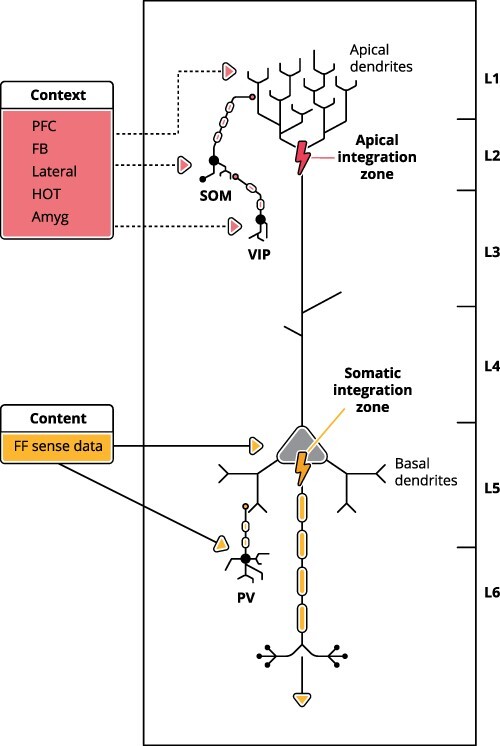
A schematic depiction of the two integration zones of a mature L5 pyramidal neuron. For simplicity, the local microcircuitry has been reduced to just those inhibitory connections that are of paramount significance for the view that apical and basal dendrites are functionally distinct. The apical dendritic tree is also referred to as the “tuft.” VIP, SOM, and PV are three anatomically, neurochemically, and physiologically distinct classes of inhibitory interneurons. Other classes are not shown here. In perceptual regions, PV cells are activated by feedforward sensory data and suppress depolarization of the soma by the basal dendrites. SOM cells receive inputs from within the same region and provide a tonic, i.e. sustained, blanket of inhibition of the apical dendrites, from which a few selected cells are released by the disinhibiting effects of VIP inhibitory interneurons (Karnani *et al.* 2014). Although not shown here to keep the diagram simple, all these inhibitory interneurons receive excitatory input from the pyramidal cells that they inhibit, thus producing oscillations at various frequencies. PFC: prefrontal cortex; FF: feedforward sensory input to the basal dendrites; FB: feedback from higher perceptual regions; Lateral: lateral interactions within the same region; HOT: “higher-order” thalamus; Amyg: amygdala; VIP, SOM, and PV: types of inhibitory interneurons

Cortical processing revolves around pyramidal neurons. Of special interest are context-sensitive cells with two points of integration as exemplified by a subset of layer 5B pyramidal neurons.^2^ These cells have two distinct zones of integration—the somatic integration zone and the apical integration zone, as shown in Fig. 1. Both zones initiate active regenerative voltage-dependent transmissions. For instance, the apical integration zone initiates calcium spikes and the somatic integration zone sodium action potentials.

The somatic integration zone of a layer 5B (L5B) pyramidal neuron receives inputs from basal dendrites that feed directly into the cell body. These inputs to the somatic integration zone operate as a chain of feedforward inputs from sensors such as the retina, via modality-specific first-order thalamic relay nuclei, such as the LGN, to primary sensory cortices, and then onward through hierarchies that extract progressively more abstract features from the information provided by the sensory input. The feedforward stimuli reaching the somatic integration zone in visual pyramidal neurons contain information about color, shape, contours, motion, surface, position in space, size, depth, and so on.

The long apical trunk of a pyramidal neuron can be divided into proximal, intermediate, and distal (tuft) segments. The apical integration zone resides in the distal upper part of the trunk oriented toward the surface of the cortex, in cortical layer 1. The elongated dendritic trunk allows for the coupling of the activity of its somatic zone (close to the neuron soma in the deeper cortical layer) and apical zone (in the upper cortical layer). With this kind of structural setup, the L5 pyramidal neuron enables the interaction between diverse contextual inputs to the apical dendrites in layer 1 and content-specific input to the somatic integration zone (Larkum *et al.* 2001).

The apical integration zone receives diverse feedback inputs from higher perceptual regions and from intralaminar and other “nonspecific” thalamic nuclei, top-down inputs from prefrontal cortical regions, and inputs from nuclei in the amygdala, from parahippocampal regions, and from neuromodulatory systems (Rockland and Pandya 1979; Cauller 1995; Diamond 1995; Rhodes and Llinás 2001; Larkum *et al.* 2004, 2009; Larkum 2013, Figure I in Box 1 at 143; Ramaswamy and Markram 2015; Stuart and Spruston 2015). The activity in the apical integration zone differs from that in the somatic integration zone in one crucial respect: its role in perception is to act as a *modulator* of the strength with which information abstracted from feedforward activity is transmitted. If the net input to the apical integration zone exceeds the calcium spike initiation threshold and the soma receives feedforward evidence for the perceptual features to which it is selectively sensitive, then those features are signaled to downstream sites more effectively. This modulation leading to more effective transmission of perceptual information coded in feedforward activity is called AA.^3^

Direct intracellular recordings show that excitatory apical input that cannot by itself generate action potential output can, nevertheless, greatly affect the cell’s response to the feedforward inputs to its basal dendrites and soma (Larkum 2013; Major *et al.* 2013); these inputs specify the receptive field features to which the cell is selectively sensitive. When the net apical input is excitatory, the cell’s response to its preferred sensory feature is amplified. However, when the net input is inhibitory, due to specialized inhibitory interneurons targeting layer 1 apical tufts (Fig. 1; SOM inhibitory interneurons), that response is attenuated. When these inhibitory interneurons are themselves inhibited by VIP inhibitory interneurons, then amplification is disinhibited. This amplifying or attenuating mode of apical function is typical of normal awake perception.

If the apical integration zone receives a backpropagated spike from the soma when it is also receiving excitation from tuft dendrites in layer 1, its threshold for initiating calcium spikes is significantly reduced. These calcium spikes, or plateaus, are actively transported to the soma via the apical trunk where they amplify response to the feedforward input by converting a single somatic action potential into a brief burst of two to four action potentials within about 20 ms. This process of backpropagation-activated calcium firing, although the most studied, is not the only intracellular process by which AA may occur (for a detailed review, see Section 2.3 of Phillips 2017).

Inputs to the apical integration zone specify a broad context within which the processing of feature-specific information by the somatic zone occurs. They impart relevant updates about bodily needs, emotional states, current tasks, learned cognitive expectations, and biases, as well as information from other streams of sensory processing, both within and between modalities. For what follows, it is important to note that inputs from all the various sources are summed at the apical integration zone to compute a single value, i.e. a postsynaptic potential at the apical integration zone. This value can in no way convey information about each of the many individual components that it sums. The information conveyed by that single value is the net bias toward either depolarizing (excitation) or hyperpolarizing (inhibition) the apical integration zone given all these diverse inputs.

In contrast to the role of AA in perception, cells in perceptual regions can also be activated during imagery experiences, hallucinations, and dreams. We assume that in these cases inputs from internal sources to the apical dendrites in layer 1 do generate an action potential output, a process referred to as “apical drive” (Aru *et al.* 2020a). As outputs from these cells are normally a response to sensory input, it is to be expected that imagery, hallucinations, and dreams will generate experiences that have much in common with veridical conscious perception.

## AA and perceptual consciousness

The theory briefly reviewed in the previous section has the potential to explain four phenomena distinctive of conscious perception. In particular, the cellular mechanisms described may help to explain (i) how the cortically received sensory signals are amplified such that they become part of conscious experience, (ii) how the apical mechanism amplifies the perceptual information transmitted without corrupting it, (iii) how the local signals to be amplified are selected in the context of the organism’s current needs, goals, and emotions, as well as of the information extracted from other sensory inputs, and (iv) how the neuromodulatory mechanisms underlying the global states of consciousness and the mechanisms of selective conscious perception are intertwined at the cellular and molecular level. In this section, we will elaborate each of these points in more detail.

### A cellular conscious-status awarding mechanism operates upon unconsciously computed perceptual contents

Perceptual contents are processed in dedicated sensory-perceptual areas of the cortex. Auditory contents are processed in primary and secondary auditory regions, perceptions of motion in the V5/MT area, areas tuned for face stimuli are found in occipital and temporal cortical areas (Chang and Tsao 2017; Nestor *et al.* 2020), and so forth. Stimulus-specific activity can be generated in these regions unconsciously, however. This activity may process perceptual inputs from the low-level features such as orientation or contrast up to higher levels involving object identities and categories or word meanings (Greenwald *et al.* 1996; Kinoshita and Lupker 2003; Boyer *et al.* 2005; Dehaene *et al.* 2006; Van Gaal and Lamme 2012; Stein *et al.* 2014; Francken *et al.* 2015; Dijksterhuis and Nordgren 2016; Song and Yao 2016; Hutchinson 2019; Koivisto and Neuvonen 2020; Lucero *et al.* 2020). Evidence indicating that contents of conscious experience can be processed unconsciously and preconsciously is rich and multifaceted.^4^ This evidence has been gathered with a variety of experimental paradigms (briefly reviewed in the Appendix).

Two empirical findings are crucial for our argument. First, stimuli of which we are not conscious engage (parts of) the same sensory areas as consciously experienced stimuli (Moutoussis and Zeki 2002; Van Vugt *et al.* 2018; Pojoga *et al.* 2020; Frith 2021). That is to say, a stimulus may activate a neural response in the appropriate part of a sensory cortex without eliciting its experience. Second, the unconscious activations are differently expressed; typically, the neural response to the same type of stimulus in the unconscious perceptual condition (as measured by functional magnetic resonance imaging (fMRI), electroencephalography (EEG), and other instruments) is “weaker” (Moutoussis and Zeki 2002; Van Vugt *et al.* 2018; Fontan *et al.* 2021; Stein *et al.* 2021). Crucially, this difference in robustness of neural response to a stimulus does not require a corresponding change in the physical attributes of the stimulus (e.g. in its contrast or intensity). This is clearly demonstrated in binocular rivalry experiments (Blake 2001; Hesse and Tsao 2020) in which a physically unchanging stimulus alternates between being consciously visible and invisible.

We interpret these findings in the following way. For conscious perception to happen, additional processing to that which has been sufficient for unconscious processing of the stimulus is needed. As the main difference between preconscious and conscious perceptual contents consists in the strength of the neural response in the same sensory areas, this additional processing has to be in its nature amplificatory. The neural processes initiating the transitions of preconscious contents into perceptual experience therefore need to possess the required amplificatory capabilities.

We propose that it is the targeted amplification of relevant sensory signals what helps to turn them into experienced contents. The apical theory introduced in the “The mechanism of AA” section provides a neurobiologically realistic mechanism for this targeted amplification. Implicit perceptual contents constituted in the activity in somatic integration zones of pyramidal neurons in L5B and elsewhere remain unconscious unless amplified by the processes of AA. We may call this cellular mechanism a “conscious-status awarding” mechanism (CSAM; Bachmann 2011).^5^ That the recorded signals in the sensory sites in unconscious perceptual conditions are weaker than in the conscious conditions can then be explained with the help of this notion. Our hypothesis, consistent with experimental evidence to be presented below, is that the weaker neural response in unconscious conditions can be explained by the absence of CSAM activation (i.e. by the absence of AA), or by its insufficient involvement. We assume that for any content to become conscious content there must be at least some minimal (critical, threshold) levels of AA applied onto neural units representing that content.

The apical consciousness-awarding mechanism is not a content-specific mechanism. In normal perception, the apical input by itself does not specify the feature or object information transmitted via inputs to the basal^6^ and perisomatic synapses. Because AA does not specify perceptual contents, it is not confined to any particular sensory modality such as vision or audition. As the presumed cellular CSAM, AA is a general mechanism to be found in the cortical areas where perceptual content is represented.^7^

On the hypothesis that we advocate in this article, implicit, preamplified perceptual contents in the somatic integration zone may retain many of their qualitative attributes such as, e.g. basic features (e.g. orientation, size, contrast polarity, and color category), shape category, basic phonemes, and so on. However, although AA itself is a content-nonspecific process, implicit contents specified in the basal zone may undergo some forms of qualitative transformations due to amplification leading to conscious perception. In particular, without changing the featural or object-category attributes of the stimulus, apical effects may enhance representational salience of visual perceptual features such as subjective contrast and brightness or subjective loudness of an auditory percept. The research literature showing context-dependent variation of subjective saliency or clarity of physically invariant stimuli is rich, encompassing different sensory modalities (Bachmann 1988; Knobel and Sanchez 2009; Liao *et al.* 2016; Gelbard-Sagiv *et al.* 2018; Itthipuripat *et al.* 2019; Carrasco and Barbot 2019; Eklund *et al.* 2020). Importantly, a change in subjective representational salience of features such as subjective contrast or brightness is just a by-product of “internal,” contextually driven amplificatory processes in the pyramidal cells. Such an enhancement does not strictly require a corresponding change in the external stimulus, in contrast to theories of perceptual consciousness such as Zeki and Bartels (1999).^8^

As a hypothesized cellular part of the CSAM, AA is distributed in modality-specific sensory cortices and other cortical areas.^9^ This is in no way a disadvantage of the present account. The cellular CSAM need not exclusively converge on a single common neuron or a local processor of any kind or a single neural structure. All that matters is that “the same type” of cellular process is deployed in different sensory modalities and in distinct areas within modal sensory sites.

It is possible to envisage the employment of this common cellular process as a successive set of cycles of AA, gradually leading to a stable conscious percept. The reverse hierarchy ideas (e.g. Hochstein and Ahissar 2002; Campana *et al.* 2016) may be relevant here. Coarse, gist-related representations are activated by AA for conscious experience first, and then top-down AA-based activations directed at increasingly concrete, specific representations are adding fine features to the gist. Given the speed of feedforward and top-down signaling volleys contrasted with how long it takes for a subjective percept to become formed (about 200 ms), there is time for such multiple cycles (with bit-by-bit corrections between each cycle).

Although there is much physiological evidence for apical processes in the mammalian brain, obtaining direct experimental evidence implicating AA in conscious perception is extremely challenging. To obtain such evidence, it is necessary to record the specific transactions between distinct parts of a single neuron and correlate them with a concurrent behavior of a living animal. Encouragingly, this correlation was established in a recent study on whisker stimuli detection in mice (Takahashi *et al.* 2016). The authors performed a two-photon imaging study of apical Ca^2+^ activity in L5 pyramidal neurons during a perceptual task. The animals were trained to signal the detection of small whisker deflections by licking to obtain water rewards. The experimental results indicate that the ability of the animal to detect whisker deflections is closely correlated with increased levels of calcium currents in the apical dendrites of L5 pyramidal cells in the primary somatosensory cortex. Inversely, the absence of reported detection correlated with a decrease in apical calcium currents. To the extent that overt discriminative behavior of an animal depends on conscious perception, the positive correlation between AA in relevant parts of the somatosensory cortex and successful behavioral detection implies that AA contributes to perceptual consciousness. In the near future, more data of this sort could be obtained with ultra high-resolution fMRI (Larkum *et al.* 2018), which can resolve neuronal activity across cortical layers and differentiate somatic from apical contributions within individual cells.

Furthermore, Takahashi *et al.* (2016) successfully applied the injunction to causally intervene into the hypothesized cellular mechanism and track observable behavioral effects (Bickle 2006). Their pharmacological and optogenetic interventions indicate that the detection threshold can be raised or lowered by enhancing or opposing active currents in the apical dendrites, thus demonstrating the causal role of such currents in conscious detection.^10^ Another intervention-based paradigm suitable for studying relations between perceptual awareness and AA is transcranial magnetic stimulation. Murphy *et al.* (2016) demonstrated that a single-pulse transcranial magnetic stimulation on dendritic activity in layer 5 pyramidal neurons of the somatosensory cortex inhibited dendritic Ca^2+^ activity. The specificity of such targeted interventions is promising. Recently, it has been convincingly argued that during general anesthesia, apical and somatic zones of pyramidal neurons are decoupled, which prevents the effects of AA (Suzuki and Larkum 2020). As a consequence, feedforward sensory processing continues to some extent during general anesthesia but cannot be brought to the level required for perceptual consciousness. A disadvantage of general anesthesia is that it abolishes all perceptual experience. Studies using transcranial magnetic stimulation could noninvasively uncover local, fine-grained ways in which apical dendrite activity in sensory regions contributes to conscious perception.^11^

### AA distinguishes modulation from the specification of the informational content

The cellular CSAM must be capable of amplifying selected sensory signals without corrupting their informational content. Feedback from higher regions is a notable source of modulatory input, but the information fed back does not become part of the sensory input to which the cell is tuned. If information from the recurrent apical inputs was merged with the cells’ somatic inputs, the message that the cell initially “broadcasts” would be lost or distorted. In L5 pyramidal neurons, this amplification without merging is achieved by the functional specialization of the two distinct integration zones.

The view of pyramidal neurons as two-point processors is in contrast with the standard view of a one-point neuron. The central tenet of the system-level neuroscience has long been that, for neurons in general, all positive and negative inputs to the soma are summed, and an action potential is triggered if that sum exceeds a threshold. However, pyramidal cells with two points of integration operate in a fundamentally different way. They use a summed input to the tuft dendrites to amplify response to the somatic and perisomatic inputs. In this amplifying mode of function, the cell’s action potentials transmit information specifically about the somatic/perisomatic sum but not about the apical sum.

Using recent advances in the foundations of information theory, amplification has been formally defined as the use of one subset of inputs to modulate the transmission of information about other inputs without transmitting any information specifically about itself (Kay and Phillips 2020). The two-point neuron view thus allows a clear distinction between modulatory amplification and merging (or “combining”) information from the somatic and apical inputs.^12^

In contrast to the Dendritic Integration Theory (Bachmann *et al.* 2020; Aru *et al.* 2020b), the hypothesis presented in this article does not require that the cellular processes comprising AA achieve a “representational match” in the content of both integration zones. This would be difficult to achieve, because the modulating apical inputs from higher regions bring a different sort of information than the feature-specific cells in the sensory regions—remember that, e.g. the amygdala is included in the diverse sources that provide inputs to the apical dendrites in layer 1. Furthermore, in order for the representational match between the two integration zones to occur, the cells in lower regions would have to inherit the large receptive fields of cells in the higher regions providing the apical feedback inputs. This, however, does not happen. All that matters for AA is whether the net excitatory input to the apical integration zone is positive when the net somatic or perisomatic input is positive. It would thus be misleading to describe the apical inputs as “matching” the somatic inputs in any sense other than in the statistics of their co-occurrence.

### Context-sensitivity and selectivity of AA

AA is not a mechanism blind to the current context of the organism’s percepts, needs, threats, or emotional state. On the contrary, AA enables rapid, effective, and energetically low-cost context-sensitive selection of information. The aim of contextual guidance in perceptual processing is to amplify transmission of information that is coherent, relevant, and informative, while attenuating transmission of other information. Apical dendrites are in this sense selective and context-sensitive. This selectivity and context-sensitivity is made possible by the diversity of the sources of apical input. As has been remarked, these inputs arrive not just from various cortical sites but also from “nonspecific” thalamic nuclei and from the amygdala. The fluctuating levels of arousal add further forms of context-sensitivity, which either increase or decrease the probability of perceptual experience and either expand or narrow its focus. AA thus combines holistic aspects of processing with local specificity at the level of individual cells.

It may be thought that some forms of perception require no context. However, on closer inspection, this assumption is problematic. Perception always occurs in the context of other stimuli, particular states of mind, and usually also in the context of particular goals. This implies that there is always the possibility that perception could be modulated at the cellular level by any aspect of that rich context. This concerns also the highly artificial and seemingly contextless types of perception, such as those present in binocular rivalry conditions. To be presented with two mutually inconsistent images, each to a different eye, already provides a very specific context of stimulation: two perceptual interpretations of what is seen are possible. In this case, two competing candidate percepts at the preconscious level resolve their competition by means of inhibitory interneurons. These interneurons are influenced by stochastic alternation of relative strengths of inhibitory presynaptic effects on layer 5 neurons representing the competing objects (e.g. in V1 or in the temporal cortex; see Theodoni *et al.* 2011; Mentch *et al.* 2019). As shown in Fig. 1, apical dendrites receive inhibitory inputs, e.g. via SOM-expressing interneurons, from which a dynamically selected few are released by the disinhibitory effects of VIP-expressing inhibitory interneurons. The evidence that is now available concerning the effects of these inhibitory/disinhibitory dynamics provides strong support for our conception of a functionally distinct apical zone (Schuman *et al.* 2021). We can assume that in the case of binocular rivalry those dynamics ensure that at any moment only one of the alternative percepts is selected for amplification and consequently becomes part of conscious experience.

The notion of perception in the absence of any context is also helpful when viewed from a different perspective. Suppose one asks, not what happens when no context is present, but what happens when contextual input received via the apical dendrites is prevented from affecting the cell’s generation of action potentials. As noted in the “A cellular conscious-status awarding mechanism operates upon unconsciously computed perceptual contents” section, this is what is thought to happen during general anesthesia: apical and somatic zones of pyramidal neurons are decoupled, which prevents the effects of AA (Suzuki and Larkum 2020). The outcome is the complete loss of conscious experience. The same seems to be true for slow-wave sleep (see, e.g. Phillips *et al.* 2018; Aru *et al.* 2020a).

**Figure 2.**
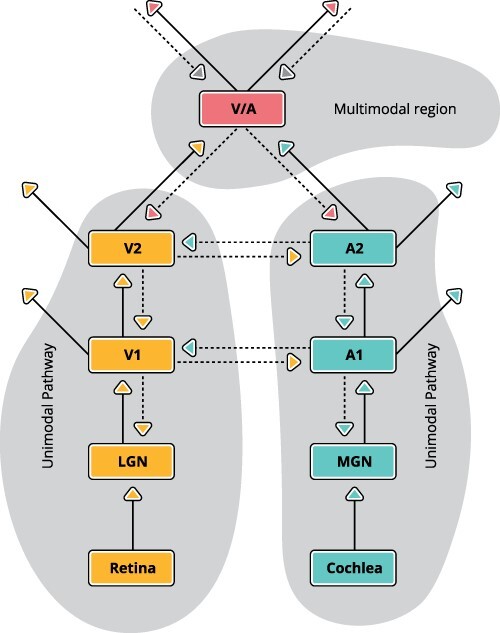
Contextual interactions between unimodal sensory regions. Direct evidence for both contextual modulation and its dependence on apical dendrites in layer 1 of the neocortex is provided by cross-modal interactions between primary sensory regions that transmit unimodal information. V1 and V2, in dark yellow, are unimodal visual regions. A1 and A2, in turquoise, are unimodal auditory regions. V/A, in pink, is a multimodal region that combines visual and auditory cues to compute supramodal abstractions. V1 and A1 transmit unimodal information to the secondary unimodal regions but are modulated by cross-modal interactions. Driving inputs from which information is extracted for feedforward transmission are shown as solid lines. Modulatory interactions are shown as dotted lines. The cross-modal modulatory interactions do not corrupt the unimodal information transmission that they modulate. LGN: lateral geniculate nucleus; MGN: medial geniculate nucleus

A striking example of contextual modulation at lower levels of perceptual hierarchies is provided by anatomical and physiological studies of the interactions between primary sensory regions. The primary sensory regions mutually interact via synaptic connections in layer 1 of neocortex (Ibrahim *et al.* 2016; Deneux *et al.* 2019, as shown in Fig. 2). These interactions, which we take to be an example of context-sensitive AA, use information from other modalities to amplify transmission of modality-specific information. For instance, such interactions can increase the detectability of a near threshold visual event if it occurs at the same time as an auditory event. The sound-induced flash illusion (Shams *et al.* 2000) is another example of this phenomenon. In sound-induced flash illusion, a single visual flash accompanied by two auditory tones is erroneously perceived as two flashes or two visual flashes accompanied by a single auditory tone are erroneously perceived as one flash. Analogous interactions occur between all combinations of primary visual, auditory, and somatosensory cortices (Meijer *et al.* 2019).^13^ Crucially, in line with what has been said in the “Apical amplification distinguishes modulation from the specification of informational content” section, these cross-modal interactions between primary sensory regions do not corrupt the unimodal information transmission that they modulate.

However, where they have been thoroughly studied, the effects of contextual modulation have been found to be a mixture of amplification and suppression that depends on many different variables in complex ways. Amplification via apical dendrites is strictly restrained by inhibitory interneurons that have evolved specifically for that purpose. Amplification needs to be restrained because, as apical dendrites are key links in positive feedback loops, there is the ever-present danger of over-amplification (as shown all too clearly by various pathologies; see, e.g. Phillips and Silverstein 2003; Phillips *et al.* 2015). Indeed, physiological evidence suggests that more interactions between primary sensory regions may be suppressed than amplified.^14^ In order for apical activation to have its amplificatory effects, specialized disinhibitory interneurons must be activated in just those pyramidal cells whose outputs are particularly relevant in the current context.

As noted, the contextual cellular processes we describe are not limited to intracortical interactions. Amplification requires the apical integration zone to be coupled to the soma, and this coupling in turn partly depends upon interactions between the neocortex and the higher-order thalamus.^15^ Specific regions of the higher-order thalamus (such as the pulvinar, posterior nucleus, and the intralaminar nuclei) send signals to both the apical trunk and the apical tufts, while it receives descending information from higher cortical regions and ascending information traveling from the receptors via collaterals bypassing specific relays. When these ascending and descending inputs are both excitatory, nuclei in the higher-order thalamus enhance informational selectivity by sending excitatory signals to the apical dendrites of pyramidal cells or to inhibitory or disinhibitory interneurons in sensory regions of the neocortex. This further enhances the signal-to-noise ratio by amplifying the response of cells that track currently relevant features, while attenuating the outputs of those that detect currently irrelevant features (Rubio-Garrido *et al.* 2009; Groh *et al.* 2014; Roth *et al.* 2016).^16^ At a more general level, ongoing thalamocortical interactions mediated by the “nonspecific” thalamic nuclei, in particular, the intralaminar nuclei, are a prerequisite for sufficient arousal, allowing context-dependent cortical activity to take place at the level accompanied by subjective awareness (Jones 2012). These thalamocortical interactions can be suppressed, e.g. by experimentally disabling the central medial nucleus of the thalamus, thus preventing it from having its typical cortical arousal effect (Timic Stamenic *et al.* 2019). A new comprehensive review of these issues by Shepherd and Yamawaki (2021) provides compelling evidence that diverse aspects of conscious perception arise from recurrent cortico-thalamic interactions in which the apical dendrites of layer 5 pyramidal neurons play a leading role.

Many inputs to layer 1, where the apical tufts of pyramidal neurons in layers 2, 3, and 5 are located, come from sources in other brain regions. Only about 10% of inputs reaching the apical zone come from nearby neurons (Binzegger *et al.* 2004). AA-centered theory therefore claims that while some feedback to the apical zone will be more local (lateral), other forms of feedback will be more long distance. This creates a time frame required by the latencies of the so-called postdictive perceptual phenomena. On some accounts, unconscious perceptual information is maintained and can be manipulated by masking up to 450 ms from the stimulus onset (Drissi-Daoudi *et al.* 2019). The present theory is able to accommodate such processing delays. It all depends on from where the signals to the apical tufts are arriving, including how many synaptic links there are between the neural source and neural target. Feedback from more remote areas such as the dorsolateral prefrontal cortex (DLPFC), frontal eye fields (FEF), or ventrolateral prefrontal cortex (VLPFC) takes time; this holds also for pathways originating from subcortical structures in the brain stem, including the intralaminar thalamic nuclei or locus coeruleus, and projecting to rostral parts of the cortex (from where the top-down signals are sent to caudal sensory-perceptual areas). All these implies that the time differences with which different kinds of percepts become conscious may depend on the localization of a source sending information to the apical inputs.

It is important, however, to bear in mind that the major part of firing responses to afferent input from receptors, which arrives at the sensory content–representing parts of L5 pyramidal neurons, have the poststimulus delay equal to 50–100 ms or less (Brazier 1977; Lamme and Roelfsema 2000; Takahashi *et al.* 2016). On the other hand, higher-level cognitive and nonspecific phasic arousal effects caused by stimulus presentation and setting the context for the stimulus take longer delays to manifest (around 100–250 ms) (Brazier 1977; Lamme and Roelfsema 2000; Gazzaniga *et al.* 2014). Thus, in order to observe more robust contextual effects on consciously perceived contents, the information setting the context has to be preset before the stimulus by some time. Because of this, the AA-centered account is also well-positioned to explain backward masking, subjective contrast enhancement in spatial precueing of target perception, the flash-lag effect, and similar phenomena exploiting the time shifts between somatic and apical inputs (Bachmann 2015). The preceding stimulus sets the spatial context, with the top-down or phasic arousal effect taking about 100–250 ms to reach cortical neurons. The feedforward information about the following stimulus reaches the cortex within 80 ms (±20 ms). For the temporal coincidence of context-controlled apical and target-controlled somatic signals arriving at the same layer 5 pyramidal neuron, the context-setting first stimulus has to precede the second stimulus by about 70 (±20) ms. In the case of backward masking the optimal temporal coincidence of the apical input and somatic input therefore takes place not for the preceding stimulus content, but for the succeeding (masking) stimulus content. As a result, AA optimizes the backward mask content for conscious perception, not the first presented stimulus content.

In the case of endogenous “attentional” spatial precueing, the pre-cue plays a similar role to the first presented stimulus in backward masking. However, as the spatial arrangement of the pre-cue and target help avoid masking (the stimuli are neighboring, not overlapping), and because now the target is not the first presented, but the following stimulus, instead of masking we get target facilitation. The pre-cue sets the apically directed spatial-contextual modulation in motion ahead in time and as soon as the specific input of the target arrives at L5 pyramidal neuron, the target is perceived.^17^ Similarly, target’s subjective contrast will be enhanced as the AA of the target-encoding cells would coincide in time with the strongest somatic input to these cells. In the flash-lag effect (where the time constants may be somewhat different), the preceding signals from the earlier time epochs of the presentation of the continuously changing input preset the apical process and as soon as the input from the following signals of the continuously changing input arrive at the soma of a pyramidal neuron, the respective content appears in conscious perception. However, if a stimulus is not preceded by streamed signals, such as when it is presented as an unchanging separately flashed object, apical modulation is not preset in time for this stimulus and it takes longer for the flashed stimulus to reach consciousness.^18^

Most of the evidence on contextual modulation at the cellular level has come from single-unit studies of species other than humans. Relevant evidence can also be provided by human neuroimaging, however. Although the use of neuroimaging to study these issues is in its infancy, there have already been encouraging discoveries. For example, studies using layer-specific, high-resolution fMRI show that visual information about an occluded part of the object or scene, inferred from the visible surround, peaks of the object or scene peaks in the superficial cortical layers (Muckli *et al.* 2015; Larkum *et al.* 2018). This result is to be expected if we assume that information about the visible surround is transmitted as a contextual information to apical synapses in the superficial layers. As fMRI signals predominantly reflect synaptic activity rather than action potentials and reflect inhibitory as well as excitatory synaptic currents, this further supports our claim that a major role for apical dendrites in layer 1 is to mediate modulatory and other effects of the current context.

### AA as a bridge between global states of consciousness and conscious perceptual contents

In consciousness studies, conscious perceptual contents are traditionally distinguished from the global states of consciousness, such as the mutually exclusive states of wakefulness, dreamless sleep, coma, minimal consciousness, dreams, or general anesthesia (Massimini *et al.* 2005; Owen *et al.* 2009; Hohwy 2009; Windt and Noreika 2011; Meyer 2015; Bayne *et al.* 2016; Fazekas and Overgaard 2016; Bayne and Carter 2018). While experienced contents are in constant flux, global states of consciousness change far less rapidly (although undergoing internal variations such as the various levels of arousal during wakefulness). It has therefore become common to treat the global states as mere background preconditions for consciousness of particular perceptual contents (see Chalmers 2000; Koch *et al.* 2016). As a consequence, experimental strategies for studying states and contents became largely independent.

This bifurcation of consciousness studies into two largely separate branches is problematic in light of the various systematic links between global states and contents. To begin with, despite continuous, moment-to-moment changes in experienced contents in a global state, the state always has “some” experienced content. Putting aside the “zero” states such as coma, dreamless sleep, or general anesthesia, we cannot be in a global state that has no subjectively experienced contents (see also Bachmann 2012; Bachmann and Hudetz 2014; Phillips *et al.* 2018; Aru *et al.* 2019). The global states are most convincingly distinguished from each other by first-person experiences of sensations, perceptions, feelings, or episodic memories (however dull, fragmented, or, to the opposite, vivid these contents are). In addition, some contents can initiate a transition in the global state: a pain may become so intense that the subject loses consciousness altogether, or a sudden sound, processed initially subconsciously, breaks through so as to wake up the recipient who thus acquires a state of waking consciousness. Equally important is the reverse dependence of experienced contents on global states. The global state determines the overall nature of experienced contents: there is a narrowly focused subjective content in an emergency, somewhat restricted or blurred content in mildly sedated subjects, and surreal content in hallucinogenic altered states.

Given all these mutual dependencies, a comprehensive theory of perceptual consciousness may be expected to elucidate the neurobiological links between global states of consciousness and experienced contents. Building on prior work of Bachmann and Hudetz (2014), Aru *et al.* (2019), and Bachmann *et al.* (2020), we will show that on the AA-centered theory, the distinction between experienced contents and global states of consciousness is not as hard and fast as is often assumed. Although global states are maintained by partly different neurophysiological systems than contents (mainly by subcortical structures in the brain stem and midbrain in the case of waking consciousness—see Baars 1995), there is close coordination in their mechanisms. Cellular mechanisms supporting apical function in L5 pyramidal cells act as a bridge between global states of consciousness and conscious perceptual contents.

At the cellular and molecular level, the link between global states and perceptual contents is forged in the delicate interplay between the targeted AAs described above and the more diffuse activity of various neuromodulators. The presence of an appropriate global state *per se* does not explain the presence of particular consciously experienced contents. Rather, the neuromodulators supporting the global states, in particular the global state of active wakefulness, pave the way for the AA of particular local contents without being in itself sufficient for perceptual consciousness. They enable and enhance the coupling between apical and somatic integration zones (Phillips *et al.* 2018; Suzuki and Larkum 2020).

**Figure 3.**
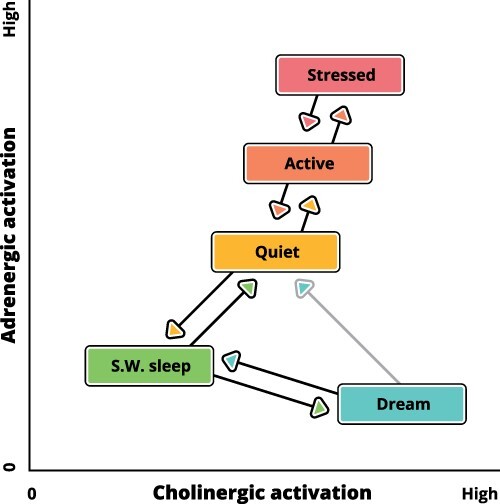
The figure shows the various levels of cholinergic and adrenergic activation that are associated with quiet, active, and stressed wakeful states and with the states of slow-wave sleep and dreaming. Activities within the apical dendritic tree and apical trunk are highly sensitive to these neuromodulators, which change the way in which these dendrites operate (as discussed in the text). Transitions between the global states can occur either gradually or abruptly. As the figure conveys only rank-order relations, the axes are in arbitrary units. The main diagonal corresponds to the activation (A) dimension of the Activation-Input-Modulation (AIM) model of the mental state (Hobson 2009). The diagonal orthogonal to that corresponds to Hobson’s modulation (M) dimension, i.e. the composite ratio of aminergic and cholinergic influences. Differences between quiet and active waking states that arise from variations in cholinergic and aminergic arousal are reported by Gervasoni *et al.* (2004). Transitions between states are based on those observed by Gervasoni *et al.*, including the infrequent transitions directly from dreaming to quiet waking. Evidence distinguishing active and highly stressed waking is reviewed by Arnsten *et al.* (2012). Evidence that cholinergic arousal is at its highest during dreaming is reviewed by Aru *et al.* (2020a)

There are significant variations in waking arousal in terms of varying degrees of alertness or sleepiness. Levels of both cholinergic and aminergic activation fluctuate during quiet, active, and stressed waking, with adrenergic fluctuations being on a faster time-scale (Fig. 3). Apart from them, differences in the degree of focus, emotional tone, and the relative dominance of external percepts and internal thoughts all co-characterize the currently dominant global state. Adrenergic, cholinergic, and other groups of neuromodulators converge on apical function as the final common pathway by which they affect the global state of consciousness. Apical dendrites are exceptionally sensitive to the neuromodulators that regulate phase changes in global states, such as those from sleep to wakefulness, from sleep to dreaming, or from quiet to active wakefulness (Carr *et al.* 2007; Barth *et al.* 2008; Robbins and Arnsten 2009). Furthermore, the master regulator of the neuromodulators, orexin (hypocretin) has a direct effect on pyramidal cells; this effect plays a major role in maintaining wakefulness (Sakurai 2007).

However, the effects of various neuromodulators are not limited to maintaining the global state and its level of arousal. Cholinergic, adrenergic, serotonergic, histaminergic, and orexinergic neuromodulators associated with arousal all specifically enhance apical function by increasing or decreasing the extent to which the apical integration zone affects the somatic output of pyramidal cells. Input to the apical integration zone has little or no effect in REM and slow-wave sleep, which correlates well with the low levels of adrenergic and cholinergic activation in these global states (Fig. 3). In the awake mode, the function of the apical integration zone is usually amplifying, but the extent of amplification is modulated by the level of arousal, ranging from moderate in states of quiet wakefulness to excessive when stress is too high. Arousal is thus a second-order contextual effect: it modulates the local contextual modulation in apical dendrites.

How is the effect of arousal on the global state of consciousness implemented in the apical integration zone? Activating adrenoreceptors in awake mice increases calcium currents in the apical tuft and lowers the threshold for the generation of amplificatory calcium spikes by the apical integration zone (Labarrera *et al.* 2018). These effects are produced by reducing the “leaking” *I*_h_ (hyperpolarization-activated) currents that flow through nonsynaptic HCN (hyperpolarization-activated cyclic nucleotide–gated) channels in apical dendrites and reduce AA. Besides adrenergic neuromodulators, histamine and serotonin also inhibit the AA-reducing *I*_h_ currents.

To repeat, this is not to say that the neuromodulatory influence itself is sufficient for perceptual awareness. Rather, it paves the way for the amplification of particular contents. Take cholinergic influence on apical function as an example of this process. Cholinergic activation is an enabling factor in apical function because it has a role in linking apical input to the soma and in disinhibiting apical input. In addition to this, cholinergic arousal increases apical effects on the cell’s output by enhancing activation of the VIP interneurons that inhibit the tonic activity of SOM inhibitory interneurons in locally specific ways (Brombas *et al.* 2014; Karnani *et al.* 2014). However, cholinergic activation itself is not sufficient to specify what will be consciously perceived: this depends on excitatory inputs to disinhibited apical branches. Without such inputs, the implicit perceptual contents processed in somatic parts of sensory neurons will not be amplified to the level sufficient for conscious perception, no matter how significant the cholinergic contributions will be.

Furthermore, as there are multiple neuromodulatory routes by which apical function can be enhanced, some of those routes may not even be necessary for normal perceptual awareness. When one of them fails, e.g. as does the adrenergic system in cataplexy, the others are still adequate to preserve perceptual experience. This is true especially of the highly correlated activities of aminergic (adrenergic, serotonergic, and histaminergic) systems. Each of them is an enabling factor that contributes to arousal that is a precondition of conscious experience, even though none of them is by itself individually necessary.

Finally, the effects of neuromodulators are not limited to their contribution of supporting and maintaining the global state of consciousness. They can also directly influence various aspects of conscious perception. For example, transient adrenergic bursts in the awake state directly increase the focus of conscious processing on selected stimuli in a way that enhances perceptual awareness of these stimuli, as well as memory for them (Mather *et al.* 2016). Similarly, drugs that increase adrenergic activation increase people’s ability to detect and discriminate weak visual stimuli; drugs that decrease adrenergic activation have the opposite effect on perception (Gelbard-Sagiv *et al.* 2018; for a mechanism with a similar effect in rodents, see Labarrera *et al.* 2018). Moreover, in some global states, the nature of consciously perceived contents is directly affected by neuromodulators. Serotonin has strong effects on phenomenology and so do the various hallucinogens associated with altered states of consciousness, such as psilocybin, muscarin, and others (Corlett *et al.* 2019; Császár-Nagy *et al.* 2019; Dos Santos and Hallak 2020; Vollenweider and Preller 2020). In sum, the distinction between global state mechanisms and content mechanisms becomes somewhat blurry at the level of intracellular processes.

## Miscellaneous issues that arise

### Relations of the AA theory to the dominant network-level accounts of perceptual consciousness

In looking for cellular foundations of perceptual experience, we do not assume that perceptual experience occurs at the cellular level (but see Edwards 2005; Sevush 2006). Neither do we claim, as Bickle (2006) does, that the network-level processes studied by the cognitive neuroscience of consciousness are a merely heuristically useful stage of inquiry, ultimately leading theorists to explanations of perceptual consciousness at the cellular and molecular levels. Rather, our claim is that events at the network level need to be complemented by explanations of cellular processes such as those described in this article, if we are to understand and explain perceptual experience. Our hypothesis is that AA provides cellular mechanisms that are necessary for the network dynamics of which our full-blown human perceptual experience consists.

In line with this proposal, AA may be conceived as the first, cellular stage of the entire coordinated set of CSAMs that turn unconscious contents into conscious ones and span multiple levels of functional organization. The AA-based theory is thus not a direct competitor of network-level theories of consciousness such as the recurrent processing theory (RPT; Lamme 2010), the global neuronal workspace theory (Dehaene 2014; Mashour *et al.* 2020), or the integrated information theory (IIT; Tononi and Koch 2015). Rather, together with (Aru *et al.* 2020b), we propose that it provides cellular mechanisms for basic capabilities implied by these theories.

On RPT, sensory areas need recurrent inputs from higher areas in order for conscious perception to take place. Apical dendrites are implicated in this recurrence on anatomical grounds because they are the dendrites most affected by recurrent signals. Recurrent signals affect apical dendrites both directly via excitatory synapses and indirectly via the activation of the interneurons that inhibit or disinhibit them. Apical dendrites are also implicated in recurrence on the grounds that recurrence is in danger of generating runaway overexcitation if activity of cells in each part of the loop is sufficient to generate activity in the next. This danger can be overcome if at least at one stage of the loop the recurrent connections cannot drive activity by themselves but operate as amplifiers of response to input from outside the loop. Importantly, on the present account, it is not recurrence *per se* that contributes to make the perceptual contents conscious. Rather, recurrence contributes to conscious perception by triggering intracellular amplifications that greatly strengthen the effects of the cell’s output at the sites to which it projects.^19^

Global neuronal workspace theory proposes a different network-level consciousness-awarding mechanism than RPT. It suggests that unconsciously encoded signals from sensory-perceptual nodes must be widely broadcast to other parts of the cortex. This idea receives some support from neuroimaging evidence that a sensory input activates more cortical regions when it is experienced than when it is not. AA may contribute to the broadcasting on which this theory relies. The output of some pyramidal cells may be “broadcast” in the sense that it is used as part of the contextual input to cells in different cortical regions. This sort of broadcasting may have a wide range of different breadths. Some pyramidal cells could provide modulatory apical input to a few other cells. Some could provide inputs to many more. No pyramidal cell could directly provide apical input to all columns in the same or other regions, but some cells may be able to contribute to the apical input of many other cells indirectly via a chain of intermediary loops. We propose that AA makes this kind of broadcasting feasible because it allows cells to receive broadcast signals while retaining their own locally specific contribution to the transmission of sensory information. The broadcast tells them whether the highly specific sensory information that they are designed to detect is or is not relevant to the ongoing activity of the system as a whole. This information about relevance is communicated to them via their apical dendrites in layer 1.^20^

There is also overlap between context-sensitive selective AA and IIT. The AA hypothesis was formulated because it provides cellular foundations for a long-lasting theory of the contextual guidance of processing and learning in the neocortex, i.e. the theory of Coherent Infomax. Although the mathematical formulation of AA as a part of the theory of Coherent Infomax is not emphasized in this article, the objectives, learning rules (Kay and Phillips 2011), and short-term activation dynamics (Kay and Phillips 2020) of that theory have all been formulated explicitly in information theoretic terms. The theory of Coherent Infomax was related in detail to cortical computation long ago (Phillips and Singer 1997), although not at the cellular level until recently (Phillips *et al.* 2016; Phillips 2017). The distinction between receptive fields and contextual fields, on which the theory of Coherent Infomax is based, is equivalent to that between the basal input, which specifies receptive field sensitivity, and apical input, which conveys contextual field information.

Thus, AA provides a cellular mechanism for the short-term dynamics of contextual modulation required by Coherent Infomax. That theory and IIT are both explicitly formulated in information theoretic terms and “information” has the same meaning in both theories. The difference is that although “Coherence” is in some ways analogous to the holistic property referred to as “Integration” in IIT, the Coherent Infomax does not conceptualize the processing in visual and other sensory areas in terms of integration: the contextual field information processed via apical dendrites does not integrate with the feedforward somatic information. In addition, IIT mostly confines the processes implementing visual perceptual experience to the “posterior HOT-zone” comprising temporal, parietal, and occipital areas (Koch *et al.* 2016). Although the AA theory also emphasizes the role of posterior areas in conscious perception, it insists that they must be supported by contextual apical inputs that partly originate beyond the posterior region. Without such inputs, perceptual contents will not be amplified and, consequently, will not become conscious.^21^

### Different kinds or degrees of AA?

A hypothesis worthy of further exploration is that different kinds or degrees of selective AA are at the root of different modes of conscious perception. For example, it is possible that information that is amplified to a small extent above the low level of spontaneous cellular activity helps to establish the broad field of conscious experience, whereas the focus of conscious experience is related to the activity of a small subset of cells, the output of which is amplified far more by attention.

A perspective on these two stages of perceptual awareness that resonates with the capabilities of context-sensitive pyramidal cells is proposed by Lamme (2004b, 2020). He interprets much of the evidence from studies of vision as indicating that there are major transitions from being fully invisible to being visible, and, from being unattended to being attended when visible. Context-sensitivity of two-point neurons could provide a cellular foundation for selection at both stages. Preattentive amplification is likely to depend on many contextual interactions that are highly local in space and time, operate in parallel across the visual field as a whole, and remain largely, if not wholly, confined to particular sensory areas. Attentive amplification in the second stage is more likely to depend on a wider context that includes current goals and emotional reactions as well as stimulus salience. It therefore depends more on nonlocal apical inputs coming from distant sources and takes longer.

As we stressed (in the “Context-sensitivity and selectivity of AA” section) the role of higher-order thalamic nuclei in context-sensitive perception, it is important to add that higher-order thalamus also participates in the control of the AA-mediated attention. As Schmitt *et al.* (2017) have recently shown, recurrent loops connecting the higher-order thalamus with regions of the prefrontal cortex (PFC) sustain attention by their amplifying effects on cortical connectivity, and are particularly complex, even in mice. Apical dendrites have a central role in these recurrent loops, with connections from the dorsal thalamic nucleus going to apical synapses near the apical spike initiation zone of cells in PFC, and connections from the ventral thalamic nucleus going to the more distant tips of the apical tuft (Collins *et al.* 2018). The functional consequences of these complexities are yet to be unraveled, but it is already clear that these recurrent corticothalamic connections avoid creating strong loops because higher-order thalamus has modulatory, rather than driving, effects on the PFC (Collins *et al.* 2018). This is in line with the finding that too much of attentional amplification may actually “extinguish” conscious awareness (see Bachmann and Murd 2010; Bachmann 2011; Murd and Bachmann 2011). Therefore, a simple picture in which more amplification always implies more sharply focused attentive awareness must be resisted.

### AA and backpropagation algorithms

Given the extraordinary success of machine learning based upon the backpropagation algorithm, the possibility that something similar to it happens in the neocortex is now under intense investigation. One of the models employed in this project is based on the distinction between apical and basal inputs (e.g. Sacramento *et al.* 2018). We agree that research on this issue is of great importance, but think that it still has far to go before it reaches the multitasking context-sensitive flexibility of the mammalian neocortex. First, backpropagation algorithms use the backpropagated signals to guide learning, not ongoing processing. In contrast to that, there is ample evidence that the neocortex uses context not only for learning but also, and primarily, to amplify the transmission of selected signals, such as those signaling the presence of a figure against the ground or providing information relevant to the current task (see, for instance, Lamme 2004a; Gilbert and Sigman 2007; Phillips *et al.* 2010). Second, analogies between backpropagation in machine learning and pyramidal cells with two points of integration face the problem of distinguishing the effects of the backpropagated input from that of all the other inputs to the apical dendrites of pyramidal cells. Third, input to the apical dendrite has been directly shown to guide learning as well as processing, but nothing in this evidence in any obvious way suggests that this process is analogous to the backpropagation machine-learning algorithm (Doron *et al.* 2020). Fourth, although attempts to develop useful algorithms in which contextual input to apical dendrites guides both processing and learning are in their infancy, there have already been some success. One of these, argued to be relevant to consciousness studies, uses the formal distinction between receptive and contextual fields to illuminate multisensory audiovisual speech processing (Adeel 2020). An earlier theory of learning with two sites of synaptic integration has highlighted the transfer only of the relevant information from higher to lower regions, with relevance being signaled by the apical input (Körding and König 2000). This theory shows how location invariant object recognition can be achieved by this intracellular mechanism. Finally, Haga and Fukai (2018) develop a model in which apical input guides fast robust learning of sequences of neuronal activity. In this model, apical input affects current processing via a multiplicative gain modulation. None of these recent models use the backpropagation learning algorithm. The consequences of such new lines of research on the contextual guidance of both learning and processing for our understanding of the neocortex, and for AI technology, are potentially groundbreaking.

## Concluding remarks

The perspective outlined in this article is essentially in agreement with the dendritic integration theory of Aru *et al.* (2020b) and Bachmann *et al.* (2020), but there are differences. First, there is a simple matter of terminology. The phrase “apical amplification” makes clear that the perspective advocated here concerns apical dendrites in particular, not dendritic computation in general. Second, and more importantly, “amplification” contrasts with “integration” in that the key point of the notion of amplification is that information about the amplifier is kept separate from the information transmitted by the signal that is amplified. Thus, the notion of amplification is explicitly distinguished from what is meant by “integration” in mathematics, and as intended in the much used description of the “integrate-and-fire” mode in which point neurons operate.^22^ Third, as noted in the “Apical amplification distinguishes modulation from the specification of informational content” section, our view of AA does not imply any form of pattern matching between apical and somatic contents. Fourth, the perspective advocated here explicitly argues that context-sensitive selection between alternatives is a cardinal function of conscious experience. Fifth, the perspective advocated here emphasizes a range of subcortical inputs to apical dendrites that extends well beyond inputs from the higher-order thalamus and includes input from neuromodulators of the arousal systems (which are crucial to the role of apical dendrites in conscious experience, as we discussed in some detail in the “AA as a bridge between global states of consciousness and conscious perceptual contents” section). In particular, we put emphasis on adrenergic arousal, which dendritic integration theory does not mention at all. Finally, our notion of AA is distinctive in being built on the long-standing mathematical formulation of the different functions of receptive and contextual fields that were developed as part of the theory of Coherent Infomax (e.g. Phillips and Singer 1997) before the discoveries suggesting that apical dendrites may be able to function as contextual field information receptors.

As the list of these differences between AA and dendritic integration theory show, the two theories are complementary, not conflicting. Our conception of AA as a cellular mechanism for selective conscious perception also resonates with several other prior proposals (e.g. Cauller and Kulics 1988, 1991; Cauller and Connors 1992; LaBerge *et al.* 2000; Spratling and Johnson 2004; LaBerge and Kasevich 2013). These different proposals have different emphases, however. For example, whereas LaBerge and colleagues put emphasis on EEG rhythms and synchronized oscillations (LaBerge and Kasevich 2017), we put emphasis on neuromodulatory arousal. These different emphases are mutually supportive, however, not antagonistic, because the different proposals share the common conception of apical dendrites in layer 1 as a channel for the amplification of selected signals.

As context-sensitive selective amplification is such a basic cognitive requirement and context-sensitive neurons with two points of integration are so ubiquitous throughout the cortex, the perspective advocated here raises indefinitely many issues whose resolution will require the efforts of many laboratories for decades to come. Only a few of these issues have been identified in this article. Still, there are several ways in which this article advances beyond prior publications arguing that apical dendrites are a major route by which context guides processing and learning. First, the possibility that this apical route provides cellular mechanisms relevant to conscious perceptual experience is examined rigorously in this article from a theoretical viewpoint and is explicitly related to currently prominent theories of the neuronal bases of consciousness. Second, earlier articles argued for close relations between AA and conscious experience on the grounds that both depend on adrenergic arousal (Phillips *et al.* 2016, 2018). This claim is modified here by increasing emphasis on the role of other neuromodulators also and cholinergic arousal in particular (see Fig. 3). Third, for the first time, cross-modal interactions between unimodal cortical regions are shown to provide clear demonstrations of both contextual modulation and its dependence on apical dendrites in layer 1 of the neocortex (Fig. 2). Fourth, AA is here related to experiments exploring the extent of nonconscious perceptual processing, such as the studies of visual masking.

Research on dendritic computation is in its infancy and still has far to go. Many will assume that an adequate computational explication of how conscious experience at the network level arises from intracellular processes is a far distant milestone in that journey. Nevertheless, it may well be reached within this decade or the next. It is already clear that functional distinctions between apical and basal dendrites have a major role in dendritic computation and that this principle has a central role in capabilities restricted to the wakeful state, such as perceptual disambiguation, attention, working memory, and learning (Poirazi and Papoutsi 2020). There is also clear evidence for distinctively human forms of apical function because the apical integration zone is more effectively separated from the soma in human pyramidal cells (Beaulieu-Laroche *et al.* 2018; Gidon *et al.* 2020), and a newly discovered inhibitory interneuron not observed in rodents is part of the inhibitory/disinhibitory microcircuitry that specifically regulates the activation of apical dendrites (Boldog *et al.* 2018). Further properties of human pyramidal cells that enable them to perform computations previously thought to require networks of neurons are reviewed by Poirazi and Papoutsi (2020). Consequences of reaching the milestone of rigorously establishing the cellular foundations of human conscious experience in explicit information processing terms are unforeseeable. They would surely be transformative for medicine by enabling strategies for managing malfunctions, whether physiological or psychological, to be engineered, rather than being developed by trial and error. The consequences of releasing the capabilities of conscious information processing from biological constraints by embodying them in silicon are at present unimaginable.

## Supplementary Material

niab036_Supp

## Data Availability

There is no data associated with this manuscript.

## Footnotes

1 We do not assume, with Bickle, that explanation of conscious perception can be completely reduced to the cellular level. We return to this point below in the “Relations of the AA theory to the dominant network-level accounts of perceptual consciousness” section.

2 It is assumed that other pyramidal neurons are also included in this class, such as some cells in layer 2/3, but the full extent of this class is not yet known.

3 See, e.g., Larkum *et al.* (1999), Ledergerber and Larkum (2010), and Larkum (2013). For an introduction to AA and its various implications in cognition, see Phillips (2017). Phillips *et al.* (2016) review the relevance of AA to global states of consciousness, with a focus on adrenergic regulation of apical function.

4 “Unconscious” means that the contents remain nonexperienced, and “preconscious” means that contents that were initially unconscious become consciously experienced at a later time.

5 In Marvan and Polák (2020) essentially the same notion is labeled “general neural correlate of (perceptual) consciousness”. Here we speak directly of the causally involved neurobiological mechanism itself (the neurobiological CSAM) and focus only on the cellular level of brain description.

6 We use “basal” and “somatic” interchangeably.

7 Generality over perceptual modalities is sought as a first step to full generality over all phenomenal contents, including the extension to emotions, thought, metacognition, dreams, working-memory contents, and so on. However, for reasons of space and because putative content representations of the higher-order cortical areas are much less known than the sensory-perceptual representations, we restrict our attention in this article to perceptual contents.

8 Here we will not delve into the otherwise important conceptual specifications of what precisely can be enlisted as qualitative attributes and what cannot. Suffice it to say that some of the stimulus (object) attributes specifying the contents may correspond to modality-specific features such as color, motion direction, shape, loudness, or somatosensory pressure, while other attributes are panmodal, such as intensity, clarity, distinctness, or pleasurableness. AA can work both as the mechanism supporting selection for consciousness of certain modally specified types of contents and as the mechanism for amplifying the saliency, including saliency as measured in terms of the panmodal attributes.

9 This does not imply that AA occurs in every part of the neocortex: there is physiological evidence that AA may occur only when the apical dendrite trunk is at least about half a millimeter long (Fletcher and Williams 2019). This is only a minor restriction on the anatomical generality of AA, though, because nearly all parts of the neocortex have pyramidal cells with trunks of that length or more, particularly in humans.

10 See also the follow-up study (Takahashi *et al.* 2020) where it is demonstrated that when the mouse’s task was simply to detect a weak whisker twitch, the effects of apical input were restricted to those layer 5B cells that project to subcortical sites.

11 These intervention-based paradigms could be supplemented with studies on the effects of pharmacological manipulations of arousal levels. Both perceptual detection and apical function are highly dependent on neuromodulatory arousal (e.g. Phillips *et al.* 2016, 2018). Further light could therefore be cast on these issues by extending pharmacological paradigms such as that of Gelbard-Sagiv *et al.* (2018), in which induced changes of arousal levels are related to perceptual detection and evoked cortical activity.

12 This feature of two-point neurons determines their role in theories of consciousness based on the notions of recurrence and broadcasting; we return to this point in the “Relations of the AA theory to the dominant network-level accounts of perceptual consciousness*”* section.

13 Further developments of this cross-modal paradigm are planned. They will focus on interactions between vision and somesthesis and will study the effects of cross-modal interactions on perceptual detection at behavioral and cellular levels in both rodents and primates (source: Johan F. Storm, private communication).

14 Widespread suppression of many cellular activities greatly increases the signal-to-noise ratio of the few that are amplified. For example, Iurilli *et al.* (2012) show that auditory information suppresses the transmission of potentially distracting visual information by V1, via the activation of specific local inhibitory circuits.

15 Whereas first-order thalamic nuclei relay sensory information from receptors to the cortex, higher-order thalamic nuclei predominantly mediate information transmission between cortical areas. The distinction between the first- and higher-order thalamus roughly corresponds to the distinction between “core” and “matrix” cells (Bickford 2016). Higher-order matrix cells are especially important for our argument because, in addition to reciprocal connections to layer 5, they predominantly target layer 1 of neocortex. For further details on the anatomy and physiology of higher-order thalamus, see Guillery (1995), Sherman and Guillery (2006), and (Saalmann 2014).

16 As some psychotic disorders of perceptual experience, such as schizophrenia, involve *N*-methyl-d-aspartate hypofunction (Phillips and Silverstein 2003), it is worth noting that contextualization via apical receptors during normal perception is highly dependent on *N*-methyl-d-aspartate receptors. Detailed reviews of these issues are provided by Larkum (2013), Major *et al.* (2013), Phillips (2017), and Silverstein *et al.* (2017).

17 Subliminal spatial-attentional precueing facilitation effects show that apical modulation can depolarize the pyramidal neuron membrane and can enhance spikes, but this effect remains subliminal. The neurons carrying unconscious content become modulated closer to up state, with subsequent delayed effects on behavior. This indicates that AA needs to reach a certain critical level in order to enable conscious perception of a stimulus.

18 The above described effects capitalize on the hypothetical facilitative apical effects, but suppressive apical effects are also possible.

19 Incidentally, this feature of the apical hypothesis shields it from the “unfolding argument” recently made against causal theories of consciousness such as the RPT (Doerig *et al.* 2019).

20 According to global neuronal workspace theory, a subset of workspace neurons coding for the current conscious content is activated in the process of “ignition” launched in the prefrontal cortex, with the remainder of the workspace neurons being temporally inhibited (Mashour *et al.* 2020). Although variants of the theory mostly remain agnostic about the cellular processes associated with ignition, Moutard *et al.* (2015, 197) note that L5 pyramidal neurons with their apical integration zones provide one possible neurobiological mechanism for ignition launching.

21 IIT has been strongly criticized for implying panpsychism (Merker *et al.* 2021). In contrast to IIT, AA as a cellular mechanism for human conscious experience associates experience with a special kind of context-sensitive information processing in the neocortex (Phillips 2012), where context includes information both from within the neocortex and from multiple other sources.

22 For details of this formal mathematical conception of amplification as a form of modulation, see Kay *et al.* (2017) and Kay and Phillips (2020).

## Appendix

### Converging lines of evidence for unconscious and preconscious perceptual processing

Evidence for unconscious and preconscious perceptual processing has been gathered with a variety of experimental paradigms. These paradigms seek the fulfillment of the following two conditions: (1) a stimulus S with perceptual/cognitive/affective content that can be explicitly experienced in conscious perception when S is presented alone becomes explicitly not experienced when S is presented in a special stimulation context (designed to prevent explicit conscious experience of S), and (2) there are experimental dependent measures (including speeded discrimination, categorization, spontaneous response selection, or specific neural processing markers) such that it is possible to demonstrate the dependence of their value on the content of S.

One group of studies allowing to achieve the above-mentioned twofold aim can be brought under the general category of “masking.” A brief stimulus (typically lasting less than 50 ms) is accompanied in space and time with a different stimulus, which masks conscious experience of its companion. One of the stimuli remains perceptually subliminal. For effective masking to be achieved, typically there is close spatial and temporal distance between both stimuli (from spatial overlap to adjacency and from temporal overlap to very short stimuli onset asynchrony, e.g. between 0 and 150 ms). For example, there are well-documented masked priming effects by subliminal semantic, response-choice, affective, or visual-category primes on subsequent identification, categorization, and autonomic unconscious threat detection responses (Van Gaal *et al.* 2010; Finkbeiner and Friedman 2011; Van Der Ploeg *et al.* 2017; Avneon and Lamy 2018; Kiefer *et al.* 2019). Even if, in principle, masking may have only a partial effect whereby not all stimulus content is deprived of explicit perception and the stimulus has not become fully subliminal (e.g. something flashed, but its perceptual category could not be explicitly discriminated above chance), that part of contents that was not consciously perceived nevertheless remains subliminal.

The neural signatures of subliminal contents may remain sufficiently robust to be decoded by third-person methods. Stein *et al.* (2021) used fMRI measurements to correctly categorize subjectively invisible masked stimuli (faces or houses). Subjectively invisible stimuli activated sensory-perceptual areas specialized in content processing (Stein *et al.* 2021, Fig. 3a and b). Interestingly, when the differences between scans in subjectively invisible and objectively invisible conditions were analyzed, narrowly focused brain areas of relatively higher activity for subjectively invisible conditions were still found. In a complementary fashion, Fahrenfort *et al.* (2017) conclude that representations of stimuli that do not enter awareness nevertheless “have a neural signature that is indistinguishable from perceptually rich representations that occur for objects that do enter into conscious awareness” (Fahrenfort *et al.* 2017, 3744). Such results are especially important for our argument in the “A cellular conscious-status awarding mechanism operates upon unconsciously computed perceptual contents” section of the main text, viz., that the function of consciousness-awarding mechanisms is not to create representations of features or objects *de novo* but to amplify them to the level sufficient for conscious perception.

Animal studies capitalizing on masking have also been informative. Paired presentation of stimuli that remained out of awareness produced “Hebbian learning” in V1 neurons and facilitated the subsequent processing of these stimuli (Pojoga *et al.* 2020). These authors demonstrated that effectively masked natural images were encoded by V1 cell populations in monkeys to the extent sufficient for later content-dependent behavioral effects. When the same images were subsequently presented above the perceptual threshold, perceptual performance and neuronal sensitivity were improved relative to the control condition. Pojoga and coauthors concluded that “exposure to subthreshold, behaviorally irrelevant, stimuli in the absence of awareness improves stimulus discriminability and perceptual performance while increasing the amount of sensory information extracted by V1 population activity, and the sensitivity and precision of individual cells. Thus, subthreshold stimuli activate neuronal networks involved in perception, and this activation contributes to subsequent changes in sensory representation” (Pojoga *et al.* 2020, 8).

Methodologically close to masking are the continuous flash suppression (Tsuchiya and Koch 2005), breaking continuous flash suppression (bCFS) (Jiang *et al.* 2007), and breaking repeated masked suppression (Abir and Hassin 2020) paradigms designed to study the content-dependent effects of subliminal stimuli. The continuous flash suppression and breaking continuous flash suppression methods are a combination of binocular rivalry (see further on) and dichoptic masking where the suppressor (in the role of mask) and contentful stimulus are presented to the different eyes, while in breaking repeated masked suppression, stimulus and mask are presented to the same eye(s), but they are repeatedly presented for longer periods. Compared to masking, these extended suppression methods allow much more prolonged presentation of the stimulus (or at least its imperative content exerting subliminally driven effects) in the subliminal mode. If a stimulus breaks through its suppression earlier than some other stimulus, and if this depends on the content of the stimulus, this constitutes a strong case for subliminal content processing. For instance, using breaking repeated masked suppression to explore what will be prioritized for subsequent emergence in consciousness, Abir and Hassin (2020) replicated the standard findings from breaking continuous flash suppression where upright faces and dominant faces break through to consciousness earlier than inverted faces and nondominant faces.

Another experimental paradigm employs “bodily movements” without or in addition to verbal report or categorical response selection as a dependent measure. In this paradigm, content-dependent, subsequent response predictive eye movements for stimuli that failed to reach awareness have been identified (Kietzmann *et al.* 2011; Spering and Carrasco 2015). Content-dependent hand movements in reaching tasks, observed and measured during preconscious stages of stimulus processing, also provide empirical evidence in support of the unconscious content processing conjecture (e.g. Song and Nakayama 2009; Finkbeiner and Friedman 2011; Freeman *et al.* 2011). However, such movement-based methods are less direct compared to, e.g. awareness clarity rating methods. It may therefore be difficult to use them to disentangle cases in which unreported phenomenal experience remains present from the complete absence of phenomenal experience.

Significant effects of masked content-based search cues on correct responses and respective “ERP signatures” have been documented (Travis *et al.* 2019). Similarly, an effect of self-relevant names (pattern masked to become subliminal) on ERP markers known to be indicative of self-relevance has been found (Doradzińska *et al.* 2020).

The list of methods and approaches validating the claim that contents can be processed and represented unconsciously could be extended further. For example, there is also the effect of implicit processing of unexpected stimuli left out of awareness due to “inattentional blindness” (reviewed in Nobre *et al.* 2020). Combining no-report paradigm with “binocular rivalry,” Hesse and Tsao (2020) showed that the same set of inferotemporal neurons is involved in both conscious and unconscious coding of visual stimuli. These are but a few examples; interested readers may learn about further details in reviews of, for instance, Kim and Blake (2005), Kouider and Dehaene (2007), Rohaut and Naccache (2017), or Soto *et al.* (2019).

Evidence suggests that unconscious content-related processes are characterized by a hierarchical nature and that whether there is conscious perception or not need not be necessarily evident according to the all-or-none rule (Breitmeyer 2015; Breitmeyer and Hesselmann 2019). Some experimental methods can control earlier level brain mechanisms necessary for some types/aspects of conscious experience, and some other methods can tap specifically into the mid-level or high-level nodes in the content representing hierarchy. Moreover, contents of experience span along modal sensory features, wholistic perceptual objects, modally categorical classes, amodal imaginary contents, and abstract semantic representations. Thus, the methodological and theoretical issues of research on unconscious processing of contents are by no means solved and pertinent research continues (see, e.g. Hurme *et al.* 2017; Peel *et al.* 2018; Greenwald and Lai 2020; Stein *et al.* 2021). Despite the lack of consensus about all the subtleties of the unconscious processing, the mass of research data shows that while unconscious processing is similar to conscious processing in many respects (e.g. associates with the same specialized cortical areas), there are also conspicuous differences. The differences most relevant to our argument in the main text concern the strength of neural response in conscious and unconscious perceptual conditions.
