# Supplementary material for: Apical amplification—a cellular mechanism of conscious perception?
Source: Neurosci Conscious. 2021 Oct 13;2021(2):niab036. doi: 10.1093/nc/niab036 (PMC8511476; doi:10.1093/nc/niab036)
Supplement: niab036_Supp [file niab036_supp.zip › Appendix.docx]

**Appendix**

*Converging Lines of Evidence for Unconscious and Preconscious Perceptual Processing*

Evidence for unconscious and preconscious perceptual processing has been gathered with a variety of experimental paradigms. These paradigms seek the fulfillment of the following two conditions: (1) a stimulus S with perceptual/cognitive/affective content that can be explicitly experienced in conscious perception when S is presented alone becomes explicitly not experienced when S is presented in a special stimulation context (designed to prevent explicit conscious experience of S), and (2) there are experimental dependent measures (including speeded discrimination, categorization, spontaneous response selection, or specific neural processing markers) such that it is possible to demonstrate the dependence of their value on the content of S.

One group of studies allowing to achieve the above-mentioned twofold aim can be brought under the general category of *masking*. A brief stimulus (typically lasting less than 50 ms) is accompanied in space and time with a different stimulus which masks conscious experience of its companion. One of the stimuli remains perceptually subliminal. For effective masking to be achieved, typically there is close spatial and temporal distance between both stimuli (from spatial overlap to adjacency and from temporal overlap to very short stimuli onset asynchrony, e.g., between 0 and 150 ms). For example, there are well documented masked priming effects by subliminal semantic, response-choice, affective or visual-category primes on subsequent identification, categorization, and autonomic unconscious threat detection responses (Avneon and Lamy 2018; Finkbeiner and Friedman 2011; Kiefer *et al.* 2019; van der Ploeg *et al.* 2017; van Gaal *et al.* 2010). Even if in principle masking may have only a partial effect whereby not all stimulus content is deprived of explicit perception and the stimulus has not become fully subliminal (e.g., something flashed, but its perceptual category could not be explicitly discriminated above chance), that part of contents which was not consciously perceived nevertheless remains subliminal.

The neural signatures of subliminal contents may remain sufficiently robust to be decoded by third person methods. Stein *et al.* (2021) used fMRI measurements to correctly categorize subjectively invisible masked stimuli (faces or houses). Subjectively invisible stimuli activated sensory-perceptual areas specialized in content processing (Stein *et al.* 2021, Fig. 3a, b). Interestingly, when the differences between scans in subjectively invisible and objectively invisible conditions were analysed, narrowly focused brain areas of relatively higher activity for subjectively invisible condition were still found. In a complementary fashion, Fahrenfort *et al.* (2017) conclude that representations of stimuli which do not enter awareness nevertheless “have a neural signature that is indistinguishable from perceptually rich representations that occur for objects that do enter into conscious awareness” (Fahrenfort *et al.* 2017, p. 3744). Such results are especially important for our argument in section 3.1 of the main text, viz., that the function of consciousness-awarding mechanisms is not to create representations of features or objects *de novo*, but to amplify them to the level sufficient for conscious perception.

Animal studies capitalizing on masking have also been informative. Paired presentation of stimuli that remained out of awareness produced *Hebbian learning* in V1 neurons and facilitated subsequent processing of these stimuli (Pojoga *et al.* 2020). These authors demonstrated that effectively masked natural images were encoded by V1 cell populations in monkeys to the extent sufficient for later content-dependent behavioral effects. When the same images were subsequently presented above perceptual threshold, perceptual performance and neuronal sensitivity was improved relative to the control condition. Pojoga and co-authors concluded that “exposure to subthreshold, behaviorally irrelevant, stimuli in the absence of awareness improves stimulus discriminability and perceptual performance while increasing the amount of sensory information extracted by V1 population activity, and the sensitivity and precision of individual cells. Thus, subthreshold stimuli activate neuronal networks involved in perception, and this activation contributes to subsequent changes in sensory representation” (Pojoga *et al.* 2020, p. 8).

Methodologically close to masking are the continuous flash suppression (CFS) (Tsuchiya and Koch 2005), breaking continuous flash suppression (bCFS) (Jiang *et al.* 2007) and breaking repeated masked suppression (bRMS) (Abir and Hassin, 2020) paradigms designed to study the content-dependent effects of subliminal stimuli. The CFS and bCFS methods are a combination of binocular rivalry (see further on) and dichoptic masking where suppressor (in the role of mask) and contentful stimulus are presented to the different eyes, while in bRMS stimulus and mask are presented to the same eye(s), but they are repeatedly presented for longer periods. Compared to masking, these extended suppression methods allow much more prolonged presentation of the stimulus (or at least its imperative content exerting subliminally driven effects) in the subliminal mode. If a stimulus breaks through its suppression earlier than some other stimulus, and if this depends on the content of the stimulus, this constitutes a strong case for subiminal content processing. For instance, using bRMS to explore what will be prioritized for subsequent emergence in consciousness, Abir and Hassin (2020) replicated the standard findings from bCFS where upright faces and dominant faces break through to consciousness earlier than inverted faces and nondominant faces.

Another experimental paradigm employs *bodily movements* without or in addition to verbal report or categorical response selection as a dependent measure. In this paradigm, content-dependent, subsequent response predictive eye movements for stimuli that failed to reach awareness have been identified (Kietzmann *et al.* 2011; Spering and Carrasco 2015). Content-dependent hand movements in reaching tasks, observed and measured during preconscious stages of stimulus processing, also provide empirical evidence in support of the unconscious content processing conjecture (e.g., Finkbeiner and Friedman 2011; Freeman *et al.* 2011; Song and Nakayama 2009). However, such movement-based methods are less direct compared to, for example, awareness clarity rating methods. It may therefore be difficult to use them to disentangle cases in which unreported phenomenal experience remains present from complete absence of phenomenal experience.

Significant effects of masked content-based search cues on correct responses and respective *ERP signatures* have been documented (Travis *et al.* 2019). Similarly, an effect of self-relevant names (pattern masked to become subliminal) on ERP markers known to be indicative of self-relevance has been found (Doradzińska *et al.* 2020).

The list of methods and approaches validating the claim that contents can be processed and represented unconsciously could be extended further. For example, there is also the effect of implicit processing of unexpected stimuli left out of awareness due to *inattentional blindness* (reviewed in Nobre *et al.* 2020). Combining no-report paradigm with *binocular rivalry*, Hesse and Tsao (2020) showed that the same set of inferotemporal neurons is involved both in conscious and unconscious coding of visual stimuli. These are but a few examples; interested readers may learn about further details in reviews of, for instance, Kim and Blake (2005), Kouider and Dehaene (2007), Rohaut and Naccache (2017), or Soto *et al.* (2019).

Evidence suggests that unconscious content-related processes are characterized by a hierarchical nature, and that whether there is conscious perception or not need not be necessarily evident according to the all-or-none rule (Breitmeyer 2015; Breitmeyer and Hesselmann 2019). Some experimental methods can control earlier level brain mechanisms necessary for some types/aspects of conscious experience, some other methods can tap specifically into the mid-level or high-level nodes in the content representing hierarchy. Moreover, contents of experience span along modal sensory features, wholistic perceptual objects, modally categorical classes, amodal imaginary contents and abstract semantic representations. Thus, the methodological and theoretical issues of research on unconscious processing of contents are by no means solved and pertinent research continues (see, e.g., Hurme *et al.* 2017; Greenwald and Lai 2020; Peel *et al.* 2018; Stein *et al*. 2021). Despite the lack of consensus about all the subtleties of the unconscious processing, the mass of research data shows that while unconscious processing is similar to conscious processing in many respects (e.g., associates with the same specialized cortical areas), there are also conspicuous differences. The differences most relevant to our argument in the main text concern the strength of neural response in conscious and unconscious perceptual conditions.
